# Energy Metabolism in Human Pluripotent Stem Cells and Their Differentiated Counterparts

**DOI:** 10.1371/journal.pone.0020914

**Published:** 2011-06-17

**Authors:** Sandra Varum, Ana S. Rodrigues, Michelle B. Moura, Olga Momcilovic, Charles A. Easley, João Ramalho-Santos, Bennett Van Houten, Gerald Schatten

**Affiliations:** 1 Pittsburgh Development Center, Magee Womens Research Institute, Pittsburgh, Pennsylvania, United States of America; 2 Center for Neuroscience and Cell Biology and Department of Life Sciences, University of Coimbra, Coimbra, Portugal; 3 PhD Programme in Experimental Biology and Biomedicine (PDBEB), Center for Neuroscience and Cell Biology, University of Coimbra, Coimbra, Portugal; 4 Department of Pharmacology and Chemical Biology, University of Pittsburgh Cancer Institute, University of Pittsburgh School of Medicine, Pittsburgh, Pennsylvania, United States of America; 5 Department of Obstetrics, Gynecology & Reproductive Sciences, and Cell Biology-Physiology, University of Pittsburgh School of Medicine, Pittsburgh, Pennsylvania, United States of America; Cardiff University, United Kingdom

## Abstract

**Background:**

Human pluripotent stem cells have the ability to generate all cell types present in the adult organism, therefore harboring great potential for the in vitro study of differentiation and for the development of cell-based therapies. Nonetheless their use may prove challenging as incomplete differentiation of these cells might lead to tumoregenicity. Interestingly, many cancer types have been reported to display metabolic modifications with features that might be similar to stem cells. Understanding the metabolic properties of human pluripotent stem cells when compared to their differentiated counterparts can thus be of crucial importance. Furthermore recent data has stressed distinct features of different human pluripotent cells lines, namely when comparing embryo-derived human embryonic stem cells (hESCs) and induced pluripotent stem cells (IPSCs) reprogrammed from somatic cells.

**Methodology/Principal Findings:**

We compared the energy metabolism of hESCs, IPSCs, and their somatic counterparts. Focusing on mitochondria, we tracked organelle localization and morphology. Furthermore we performed gene expression analysis of several pathways related to the glucose metabolism, including glycolysis, the pentose phosphate pathway and the tricarboxylic acid (TCA) cycle. In addition we determined oxygen consumption rates (OCR) using a metabolic extracellular flux analyzer, as well as total intracellular ATP levels by high performance liquid chromatography (HPLC). Finally we explored the expression of key proteins involved in the regulation of glucose metabolism.

**Conclusions/Findings:**

Our results demonstrate that, although the metabolic signature of IPSCs is not identical to that of hESCs, nonetheless they cluster with hESCs rather than with their somatic counterparts. ATP levels, lactate production and OCR revealed that human pluripotent cells rely mostly on glycolysis to meet their energy demands. Furthermore, our work points to some of the strategies which human pluripotent stem cells may use to maintain high glycolytic rates, such as high levels of hexokinase II and inactive pyruvate dehydrogenase (PDH).

## Introduction

Human embryonic stem cells (hESCs) are self-renewing and pluripotent cells derived from the inner cell mass (ICM) of a blastocyst prior to implantation. However, although they can be differentiated into any somatic cell lineage, they cannot be genetically matched to putative patients in possible cell replacement therapies. However, it has been recently shown that human somatic cells can be reprogrammed into hESC-like pluripotent stem cells, the so called induced pluripotent stem cells (IPSCs), by the ectopic expression of a combination of pluripotency factors and using a variety of approaches [1], [2], [3]. The differences and similarities of hESCs and IPSCs have been studied at several levels, namely differentiation potential, global gene expression, and epigenetic status. The various analyses have revealed that despite the high level of resemblance in terms of pluripotency these cell types are not identical [4], [5], [6], [7], [8]. Namely, gene expression profiling revealed that, while IPSCs are highly similar to ESCs, a unique gene expression signature appears in IPSCs regardless of their origin [4]. Furthermore, DNA methylation analysis revealed that the methylome of certain developmental genes in IPSCs was intermediate between that found in differentiated cells and ESCs. In other cases, the methylation pattern in IPSCs was exclusive, differing from both ESCs and differentiated cells [6] , suggesting both the occurrence of abnormal methylation during reprogramming and that some “epigenetic memory” from the somatic cells reprogrammed may linger in IPSCs, with unknown consequences [9], although the extent of this phenomenon is questioned [10]. Recent data suggests that IPSCs may be more prone to genetic mutation and instability [11], [12], [13]. Recently, Zhao et al. [14] demonstrated immunogenicity differences between authentic ESCs and iPSCs. Given the potential of these cells, further studies are needed to scrutinize these differences and to understand their impact on differentiation potential and possible therapeutic applications.

The mammalian embryo resides in a hypoxic environment prior to implantation [12]. During the early stages of embryonic development there is a metabolic shift from oxidative phosphorylation (OXPHOS) to glycolysis, and oxidative metabolism is only fully reinstituted after implantation [15]. Studies in the mouse and hamster indicated that the number of mitochondria is higher in the trophectoderm (TE) than in the ICM. Furthermore, the ICM is characterized by spherical and depolarized mitochondria with low O_2_ consumption, whereas mitochondria of the TE are elongated and have both higher mitochondrial potential and higher O_2_ consumption [16], [17], [18].

Similarly to ICM cells, embryonic stem cells rely mostly on glycolysis for energy supply. Furthermore, the mitochondria in these cells are rather immature with perinuclear localization. As human pluripotent stem cells differentiate they acquire more mature mitochondria and undergo a metabolic switch from glycolysis to OXPHOS [16], [19], [20], [21]. In agreement with these findings several authors have reported that hypoxia is beneficial for the maintenance of hESCs in a pluripotent state [22], [23]. In addition low O_2_ tensions have been reported to increase the reprogramming efficiency of both mouse and human somatic cells [24].

It is known that glycolysis and OXPHOS function in a coordinated fashion, and that the former generates 18 times more ATP per molecule of oxidized glucose than the former [25]. The first glycolytic reaction is crucial as glucose is captured within the cell by conversion to glucose 6-phosphate. This is one of the rate limiting steps in glycolysis and is catalyzed by hexokinases. Cell types with high glycolytic rates have been reported to have high expression levels of hexokinases [26], [27]. Glucose 6-phosphate can be further metabolized in glycolysis producing two molecules of pyruvate, NADH and ATP. In normoxic conditions pyruvate enters mitochondria and links glycolysis to aerobic respiration by entering the TCA cycle (which provides the mitochondrial electron transfer chain (ETC) with reducing agents for ATP synthesis) as acetyl-coenzyme A. However under low O_2_ tensions or in the presence of dysfunctional mitochondria pyruvate will be converted to lactate.

The pyruvate dehydrogenase (PDH) complex, localized in the mitochondrial matrix is the link between glycolysis and the TCA cycle. It is composed of several copies of three catalytic proteins (E1, E2 and E3) that catalyze the irreversible decarboxylation of pyruvate to acetyl-coenzyme A and NADH [28], [29]. The E1-alpha subunit is considered the on/off switch of the PDH complex, as its phosphorylation by one of the four pyruvate dehydrogenase kinase isoforms (PDHK1-4) leads to PDH complex inactivation, whereas removal of one phosphate group by one of the two pyruvate dehydrogenase phosphatases (PDP1 and PDP2) activates the complex [28], [29]

The exact mechanism by which hESCs maintain an anaerobic metabolism even in the presence of oxygen remains largely elusive. However, there is certain putative parallelism with what has been described for cancer cells, which also maintain glycolysis as a key metabolic pathway under normoxia, in detriment to OXPHOS, the so-called Warburg effect [30]. Furthermore it has been recently shown that some cancer lines cultured under hypoxia acquire an undifferentiated phenotype similar to that of ESCs [31]. The aerobic glycolysis feature of cancer cells may involve the regulation of PDH function, at least in some cases, and indeed modulation of PDH function has been therapeutically considered in cancer [32]. However these analogies must be considered with great care, as there are considerable metabolic variations between cancer cell lines [33], [34].

Additionally, it would be of interest to determine if and how IPSCs acquire a more glycolytic metabolism upon reprogramming, or if they instead maintain the metabolic features of the original somatic cell type that was reprogrammed. In this study our aim was to answer these questions by further characterizing the energy metabolism of both hESCs and human IPSCs when compared with their differentiated somatic counterparts. Towards that goal we analyzed mitochondrial morphology, glucose-related gene expression, OCR, intracellular ATP levels, lactate production and protein levels of regulatory enzymes relevant in metabolism.

In accordance to previous reports our results demonstrate that hESCs have mitochondria consistent with lower activity, at least when compared to differentiated cells. Furthermore we show that mitochondria in IPSCs are morphologically distinct from both hESC and differentiated somatic cell mitochondria. In addition IPSCs are not identical to hESCs in terms of glucose-related gene expression, although they cluster with hESCs rather than with their somatic counterparts. Moreover analysis of OCR, intracellular ATP and lactate levels confirm that human pluripotent stem cells rely mostly on glycolysis to meet their energy demands. Finally our study demonstrates that human pluripotent stem cells express high levels of hexokinase II and have an inactive PDH complex. In all parameters quantified IPSCs seem to be close to hESC, but at slightly lower levels of expression/activity. These results shed light on some of the mechanisms that human pluripotent cells use to maintain high levels of glycolysis under normoxia.

## Materials and Methods

### Cell culture

In this work we used hESC lines WA07, WA09 and WA01 (WiCell Research Institute, Madison, WI), human IPSC lines HFF1 iPS, IMR-90 iPS (WiCell Research Institute) and AE iPS (derived in our laboratory). In terms of differentiated fibroblasts, both the IMR-90 human diploid fibroblast and the human foreskin fibroblast (HFF1) strains were obtained from American Type Culture Collection (ATTC, Manassas, VA); these lines were reprogrammed to form the IMR-90 iPS and HFF1 iPS lines noted above, respectively. Finally, differentiated H7TF fibroblasts were isolated from a WA07 ESC-derived teratoma, following injection of WA07 hESCs into immunocompromised mice.

HESC and IPSC lines were cultured in mTeSR™ (STEMCELL Technologies, Vancouver, BC, Canada) on matrigel (BD Biosciences; San Jose, CA) coated dishes. Both H7TF and HFF1 were cultured in CF1 medium containing: 90% Dulbecco's Modified Eagle's medium; 10% fetal bovine serum (FBS); 1% MEM non-essential amino acids; 1% penicillin/streptomycin (Pen/Strep) and 1% 2 mM L-glutamine (all from Invitrogen, Carlsbad, CA). The IMR-90 line was cultured in 90% Eagle's minimal essential medium (ATTC, Manassas, VA), 10% FBS and 1% Pen/Strep. All lines were maintained at 37°C, 21% O_2_ and 5% CO_2_. Throughout the work WA07, IMR-90 iPS, H7TF and IMR-90 lines were used in the majority of the experiments in order to compare cell lines that have the same genetic background. The other lines were used in key experiments to consolidate the results trying to avoid any artifact that could be line specific or culture derived.

### Transduction of pluripotent and differentiated cells using a baculovirus system

Mitochondria GFP labeling was performed using the organelle lights Mito reagent (CellLight™ Mitochondria-GFP by Molecular Probes/Invitrogen). This system is a baculovirus system that contains a leader sequence for the E1 alpha pyruvate dehydrogenase fused with GFP protein. Cells were plate on coverslips and cultured for three days or 24 hours, for pluripotent and differentiated cells, respectively. Cells were rinsed with PBS, and incubated in PBS containing 82% organelle lights reagent during 30 minutes at room temperature with gentle shaking. Organelle lights mixture was removed and cells were incubated with appropriated media containing 0.1% enhancer solution (provided) for two hours at 37°C. Enhancer solution was removed and replaced by cell culture media. Although the transfection efficiencies were low, 20 hours later, GFP protein could be observed in the mitochondria. Cells were then fixed with 4% paraformaldehyde (Sigma-Aldrich) and imaged using a TCS-SP@ laser scanning confocal microscope (Leica, Microsystems, Gmbh, Wetzlar, Germany).

### Electron Microscopy

Cells were cultured on matrigel or matrix free conditions for pluripotent cells or differentiated cells, respectively. Cells were rinsed with PBS and fixed with 2.5 % glutaraldehyde in PBS, pH 7.4, for 1 hour at room temperature. The fixative was removed and samples were washed 3 times with PBS at room temperature for 10 minutes each. Samples were post-fixed for 1 hour at 4°C in 1% osmium tetroxide with 1% potassium ferricyanide, followed by three washes with PBS (10 minutes each). Afterwards, samples were dehydrated using a graded ethanol series (30%, 50%, 70%, and 90%-10 minutes) with three changes in 100% ethanol (15 minutes each). Samples were then incubated three times with a 100% Epon solution (1 hour each). Finally, samples were incubated for 1 hour with Epon solution, maintained overnight at 37°C and afterwards at 60°C for 2 days. Sections were made using an ultra microtome and imaged in a 1011 CX Transmission electron microscope (JEOL, Tokyo, Japan).

### RNA extraction, DNA clean up and Glucose RT^2^ profiler PCR array

Total RNA isolation using the TRIzol reagent (Invitrogen) and genomic DNA was eliminated with DNA-free kit (Ambion, Austin, TX) as previously described [35]. An additional step of DNA clean up was performed using the genomic elimination kit from SABiosciences (Frederick, MD). In brief, 1 µg of RNA was incubated with genomic DNA elimination mixture (containing 2 µl of 5X genomic DNA elimination buffer and nuclease free water) for 5 minutes at 42°C. The first cDNA strand was synthesized by incubating the product of the genomic DNA elimination reaction with 10 µl of the Reverse transcriptase (RT) mixture (5 x reverse transcriptase (RT) buffer, RT, primers and external control, and nuclease free water) for 15 minutes at 42°C. Reactions were immediately stopped by heating at 95°C for 5 minutes. RT products were further diluted with 91 µl of nuclease free water. cDNA samples were then loaded in the glucose gene expression panel (SABiosciences), run in a 7900HT Real Time system (Applied Biosystems, Foster City, CA) accordingly to the following thermal cycle conditions; 95°C for 10 minutes, and 40 cycles at 95°C for 15 seconds and 60°C for one minute. Each array contained wells that allowed us to control for genomic DNA contamination, and first cDNA synthesis efficacy. Twelve genes (*ALDO B*, *FBP1*, *FBP2*, *G6PC*, *GCK*, *GYS2*, *HK3*, *PCK1*, *PDK4*, *PGK2*, *PKLR* and *PRPS1L1*) were not considered in the analysis, as their Ct value was above 30. Gene expression differences were calculated using the -ΔΔCt method. Gene expression was normalized to the housekeeping gene β-actin and fold change was calculated relative to the hESC line WA07. p values were determined using online software provided by SABiosciences.

### High Performance liquid Chromatography (HPLC)

Pluripotent cells were cultured as mentioned above for seven days, whereas differentiated cells were cultured for two days. Cells were enzymatically dissociated with Accutase (Millipore) or TripLe Express (Sigma-Aldrich, St. Louis, MO) for pluripotent cells and differentiated cells, respectively. Adenosine triphosphate (ATP) was extracted with 0.6 M perchloric acid (Sigma) supplemented with 25 mM EDTA-Na (Sigma) followed by centrifugation. Supernatants were neutralized with 3M potassium hydroxide in 1.5M Tris. ATP separated by HPLC. The detection wavelength was 260 nm and the column was a Licrosphere 100 RP108 5 µm (Merck). The elution buffer for was 100 mM phosphate buffer (pH 6.5) supplemented with 1% methanol. Standard for ATP was purchased from Sigma- Aldrich.

### Lactate Production

Lactate levels were determined using the Lactate assay kit (Biovision). In this assay lactate is enzymatically oxidized and the product of this reaction reacts with a lactate probe (provided) and emits fluorescence at Ex/Em = 535/587 nm. Both pluripotent and differentiated cells were plated and cultured for 48 hours. At this point, fresh media was added to the cells. Twenty-four hours later, 500 µl of medium was collected, and cell numbers were determined. Medium samples were processed following manufacturer's instructions. Fluorescence was determined using a microplate reader (Spectra Max M2; Molecular Devices, Sunnyvale, CA). Lactate concentrations were normalized to cell number.

### Western blotting

Cells were maintained on matrigel or matrix free conditions for pluripotent cells or fibroblasts, respectively. Pluripotent cells were dissociated using Accutase (Millipore), whereas for differentiated cells Triple E (Sigma-Aldrich) was used. Total cell extracts were lysed in RIPA buffer (Sigma) supplemented with 1 mM phenylmethylsulphonyl fluoride (Sigma-Aldrich) PMSF and 2x Halt phosphatase inhibitor cocktail (Pierce, Rockford, IL). Mitochondrial extracts were isolated using mitochondria isolation kit (Sigma-Aldrich), as previously described by Wieckowski and colleagues [36]. In brief, cells were washed once with PBS, and incubated on ice with the extraction buffer supplemented with cell lysis detergent (1∶200) and PMSF (1∶200) for 5 minutes. Samples were resuspended every minute. Afterwards, we added 2v/v of extraction buffer and centrifuged twice, at 4°C, for 10 minutes, at 2000 g. Pellets containing the cytosolic fraction were stored at −80°C. Supernatants were transferred to a new tube and centrifuged, at 4°C for 10 minutes, at 11000 g. Supernatants were discarded and the pellets containing the mitochondrial fraction, were washed with extraction buffer, and centrifuged at 4°C, for 20 minutes, at 11000 g. The debris fraction (supernatant) was stored, as well as, the mitochondrial fraction at −80°C. Protein quantification was carried out using the Bradford assay (Bio-Rad laboratories, Inc., CA) and 7 µg of protein were separated in 4%–15% SDS Page gels (Bio-Rad laboratories). The primary antibodies used were: anti - Oxphos complex kit (1∶500, Invitrogen); anti-PDH (1∶500, Cell Signaling and Technologies); anti- PDHK1(1∶500, Cell Signaling and Technologies); anti- phospho PDH-E1α Ser ^293^ (1∶250, EMD); anti-hexokinase II (1∶500, Cell Signaling and Technologies), and anti-Nanog ( 1∶500, Kamiya Biomedical Company, Seattle, WA), ECL Advanced Western Blot Detection kit (Amersham Biosciences, Piscataway, NJ) was used for detection.

### Immunocytochemistry

Cells were cultured on coverslips for six days or two days for pluripotent and differentiated cells respectively. Cells were fixed in 2% formaldehyde, rinsed in PBS, permeabilized with PBS 0.1 % Triton and incubated with blocking solution (containing 0.3% BSA, 1x PBS and 5% of normal serum from the specie of the corresponding secondary antibody) for an hour at room temperature. The primary antibodies used were anti-hexokinase II (1∶500, Cell Signaling and Technologies). Antibodies were incubated overnight at 4°C, followed by three washes of 15 minutes in PBS 0.15 Triton. Respective secondary antibodies were incubated for one hour at 37°C, followed by three washes in PBS 0.1% Triton. Coverslips were assembled in slides using the fluorescence mounting media Vectashield with DAPI (Vector Labs, Switzerland). Slides were imaged using a TCS-SP2 laser scanning confocal microscope (Leica).

### Oxygen consumption rates

O_2_ consumption was determined using Seahorse XF24 extracellular Flux analyzer as previously described by Qian and Van Houten [37]. Briefly, cells were seeded in the 24-well XF24 cell culture plate in the respective culture media. For pluripotent cells, matrigel coated plates were used and cells were allowed to grow for three days, whereas for differentiated cells 300 000 cells were seeded and allowed to grow for 24 h. Thirty minutes prior to the run, culture media was replaced by unbuffered DMEM and plates were incubated at 37°C for 30 minutes for pH and temperature stabilization. Three mitochondrial inhibitors; oligomycin (1 µM), FCCP (300 mM) and rotenone (1 µM) were sequentially injected after measurements, 2, 4, and 6, respectively. After all the measurements were completed, cells were dissociated and counted.

### Statistical analysis

Means and standard error of the mean (SEM) were calculated and statistically significant differences were determined by One-way ANOVA followed by Dunnett's Multiple Comparison test. n refers to sample size. Statistical significance was determined at p<0.05. The p values for the Glucose array were calculated using SABiosciences online software. The test used was a two-tailed Student's t-test, and statistical significance was determine at p<0.05.

## Results

### Mitochondrial localization and morphology

**Figure 1:**
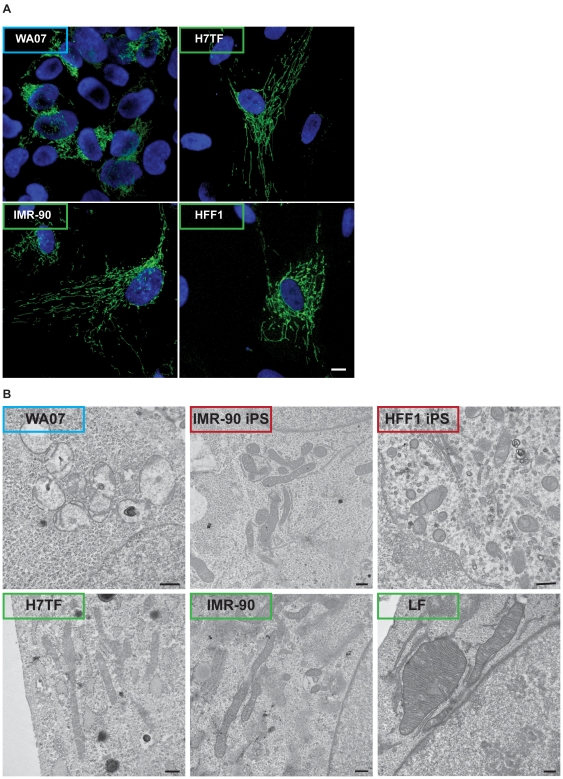
Mitochondrial morphology and localization in human pluripotent stem cells vs. differentiated cells. **A)** Transduction of hESCs (WA07 line,) and differentiated cells (H7TF, HFF, and IMR-90 lines) with organelle lights MitoGFP was used investigate mitochondria morphology and localization within the cell. Blue: DAPI; Green: pyruvate dehydrogenase GFP. Scale bar: 10 µm **B)** Transmission electron microscopy (TEM) was used to investigate mitochondria morphologic features in hESCs (WA07 line), IPSCs (HFF1 and IMR-90 IPSC lines), and fibroblasts cells (H7TF, IMR-90 and HFF1). Scale bar: 500 nm.

In order to determine mitochondria morphology and localization within the cell in pluripotent *versus* differentiated cells we transduced hESCs (WA07 line) and fibroblasts cells (H7TF, IMR-90 and HFF1) with a baculovirus system containing a leader sequence for PDH E1 alpha subunit fused with the GFP protein. Mitochondria in the hESC line WA07 showed perinuclear localization (Fig. 1A, top left) whereas the mitochondria of their differentiated counterparts (H7TF) were distributed in the cytoplasm (Fig. 1A, top right). This might be a consequence of the high nuclear-cytoplasmic ratio observed in the hESCs. Furthermore, the mitochondria in the WA07 showed a globular shape, whereas mitochondria in the H7TF line were mainly elongated and formed extensive reticular networks. Similarly, the mitochondria of the somatic lines HFF1 and IMR-90 were also distributed in the cytoplasm rather than cluster around the nucleus; their shape was mostly tubular and they formed extensive networks (Fig. 1A, bottom left and right).

In order to better characterize mitochondrial morphological features in pluripotent versus differentiated somatic cells we performed transmission electron microscopy (TEM) for hESCs (WA07 line), IPSCs (IMR-90 and HFF1 IPSC lines) and fibroblasts cells (H7TF, IMR-90 lines) (Fig.1B). TEM analysis demonstrated that mitochondria in hESCs have few cristae and electron-lucid matrix (Fig. 1B, top left). These results are in accordance with previous reports [20], [21]. TEM of cells obtained after differentiation of W07 hESCs (H7TF) revealed mitochondria with an elongated shape, and with a higher number of cristae, as well as a denser matrix (Fig.1B. bottom, left). Similar to H7TF, IMR-90 and lung fibroblasts (LF) showed mature mitochondria with numerous cristae and electron dense matrix (Fig.1B. bottom center and right, respectively). Interestingly, both IMR-90 and HFF1 IPSCs showed a mix of both elongated and globular mitochondria. Furthermore, the matrix of the mitochondria resembled that of differentiated cells (Fig.1B top, center and right, respectively). These results suggest that mitochondria morphology in human IPSCs is not identical to that found in hESCs, but IPSCs seem to have a mixed phenotype between that found in hESCs and differentiated cells. It would be of interest to perform histomorphometric analysis in order to fully validate this assumption.

### Metabolism-related gene expression in human pluripotent stem cells vs. differentiated cells

**Figure 2:**
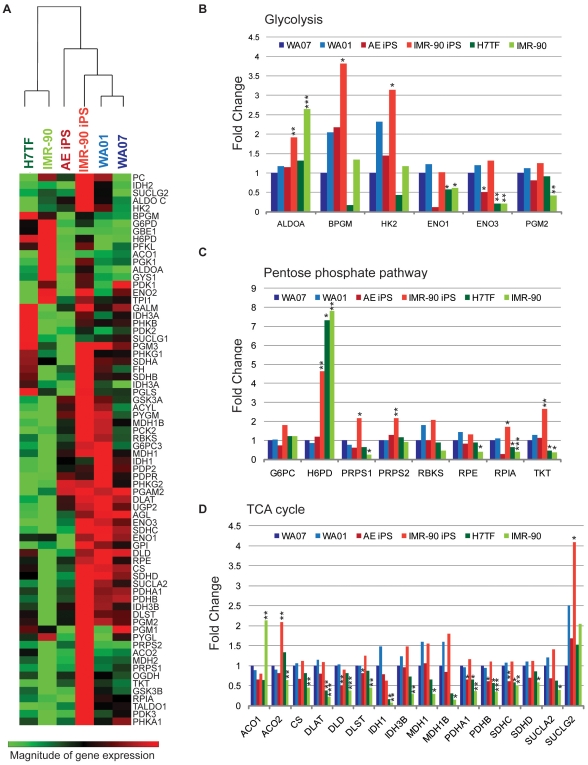
Metabolism-related gene expression in human pluripotent stem cells vs. differentiated cells. **A)** Heat map of average gene expression. Gene expression is represented as log_10_ of Ct values. An increase in gene expression is depicted in red, whereas a decrease in gene expression is represented by the green color. No differences in expression are depicted in black. Clustering was performed using SABiosciences online software. The various genes were grouped accordingly to their participation in different metabolic pathways. **B)** Glycolysis-related genes with at least two fold difference when compared to the WA07 line. **C)** Pentose phosphate pathways-related genes with at least two fold differences when compared to the WA07 line. **D)** TCA cycle- related genes with at least two fold differences when compared to the WA07 line. Fold changes were calculated using the -ΔΔCt method relative to the WA07 line. hESC lines are represented in blue, IPSC lines in red and somatic cell lines in green . The values represent means of three independent experiments. Statistical analysis was performed using Student's t test (SABiosciences online software) and significance was determined at p<0.05. Statistically significant differences are represented in Table 1.

To further investigate the metabolic pathways used by human pluripotent stem cells and differentiated somatic cells we used two hESC lines (WA07 and WA01), two IPSC lines (IMR-90 IPS and AE IPS), and two somatic lines (WA07 teratoma fibroblasts-H7TF, and IMR-90 fibroblasts) and performed the SABiosciences human glucose metabolism RT^2^ profiler PCR array. This array contains 84 key genes involved in the regulation of glucose and glycogen metabolism, along with five housekeeping genes. Overall we observed that pluripotent lines tend to cluster together rather than with their somatic counterparts (Fig. 2A, heatmap). Furthermore, we observed that there are no significant differences in gene expression between the two hESC lines (WA07 and WA01) used in this study (Table 1). Statistical analysis showed that when compared to WA07 cells IMR-90 iPSCs showed 25 genes with significantly different expression, whereas in AE iPSCs there were14 such genes (Table 1). For differentiated cells, IMR-90 cells showed 44 differentially expressed genes whereas for H7TF there were 18 (Table 1). We further focused our analysis on 60 genes known to be involved in; glycolysis, pentose phosphate pathway and TCA cycle (Fig. 2, Table 1). Due to their pleiotropic nature some of the genes were included in more than one group. In addition, for the analysis we considered genes that were differentially expressed at least two fold when compared with the WA07 line, and with p<0.05.

**Table 1:** p values of fold changes for gene expression.

	WA01	AE iPS	IMR-90 iPS	H7TF	IMR-90
Glycolytic signature in human pluripotent stem cells and differentiated cells					
GBE1	0.175241	0.182796	0.401636	0.059971	0.003255
GYS1	0.560387	0.305042	0.073519	0.361399	0.006032
UGP2	0.140358	0.583771	0.349007	**0.009063**	**0.00436**
AGL	0.716072	0.005425	0.192293	**0.003653**	**0.000895**
PGM1	0.118692	0.108458	0.557582	0.443997	0.255193
PGM2	0.314817	0.234121	0.248629	0.699586	**0.002456**
PGM3	0.219734	0.069024	0.263436	0.527696	0.591157
PYGL	0.12035	0.182944	0.63261	0.469592	0.834941
PYGM	0.105912	0.300795	0.072922	0.163273	0.161997
GSK3A	0.119705	0.144921	0.083618	0.408039	0.061973
GSK3B	0.434825	0.208689	0.086282	0.844473	0.094813
PHKA1	0.117699	**0.046949**	0.322249	0.283362	**0.011132**
PHKB	0.759246	0.227841	0.905398	0.490711	0.052339
PHKG1	0.857115	**0.004105**	0.098563	0.538787	0.712081
PHKG2	0.184464	0.330626	0.305181	0.085937	0.09916
Pentose phosphate signature in human pluripotent stem cells and differentiated cells					
G6PD	0.222918	0.12939	0.009571	0.000621	0.000546
H6PD	0.536777	0.772588	0.001435	0.022755	0.005125
PGLS	0.854679	**0.021219**	**0.088223**	0.276943	0.975412
PRPS1	0.245115	0.126676	**0.019419**	0.128065	**0.018916**
PRPS2	0.831567	**0.056076**	0.004843	0.465994	0.305017
RBKS	0.119577	0.899118	0.072344	0.913592	0.133126
RPE	0.121207	0.471102	0.264923	0.753951	**0.037438**
RPIA	0.300618	0.263414	0.010121	**0.012123**	**0.000578**
TALDO1	0.61781	**0.043175**	0.123504	**0.065059**	**0.003374**
TKT	0.152685	0.541674	0.001309	**0.020472**	**0.010954**
TCA signature in pluripotent stem cells and differentiated cells					
ACLY	0.107296	0.234368	0.061387	0.393416	0.246977
ACO1	0.399131	0.079385	0.17169	0.083256	0.001386
ACO2	0.211968	0.089029	0.006734	0.15926	**0.006435**
CS	0.598438	0.052026	0.45459	0.548219	**0.003514**
DLAT	0.23882	0.369543	0.531728	**0.001807**	**0.000145**
DLD	0.860033	**0.027963**	0.493081	0.671359	**0.004943**
DLST	0.628177	**0.019154**	0.018305	0.726826	**0.036226**
FH	0.189939	0.945714	0.123065	0.242483	0.105099
IDH1	0.112953	0.38673	0.274658	**0.0133**	**0.009346**
IDH2	0.109272	0.7786	0.031926	0.983299	0.759598
IDH3A	0.291665	0.144282	0.699497	0.35153	0.107709
IDH3B	0.126595	0.753791	0.063038	0.271417	**0.002515**
IDH3G	0.127383	0.888987	0.020491	0.160844	0.22335
MDH1	0.136175	0.888013	0.220693	0.24667	**0.032701**
MDH1B	0.162897	0.520214	0.113153	0.126206	**0.019708**
MDH2	0.741719	0.076133	0.030813	0.960944	**0.012102**
OGDH	0.20949	0.383583	0.033559	0.773533	**0.003039**
PC	0.10867	0.486329	0.070307	0.698611	0.144088
PCK1	0.606445	**0.046384**	0.011521	0.331536	0.339268
PCK2	0.10813	0.884791	0.082829	0.167213	0.250898
PDHA1	0.770854	**0.041691**	0.395379	**0.045818**	**0.00137**
PDHB	0.529193	**0.012731**	0.486834	**0.018482**	**0.000693**
SDHA	0.518039	0.053203	**0.04001**	0.445353	0.210469
SDHB	0.835404	0.151871	0.250403	0.557484	**0.01685**
SDHC	0.232507	**0.009183**	0.168632	**0.033296**	**0.002187**
SDHD	0.415562	0.059517	0.463437	0.475622	**0.011049**
SUCLA2	0.340556	0.193395	0.129939	0.172629	**0.014183**
SUCLG1	0.328695	**0.025023**	0.153357	0.198642	**0.045376**
SUCLG2	0.106127	0.465278	0.01718	0.621296	0.237038

Glycolysis; Pentose phosphate and TCA-related genes p values. p values of fold change were determined relative to the WA07 line. Statistical significance was determined at p<0.05 by Student's t test (SABiosciences online software). p values represented in red correspond to genes that are significantly up regulated, whereas p values depicted in green represent genes that are significantly down regulated. p values above 0.05 are represented in black.

Regarding the glycolytic signature, six genes among all lines were either up regulated or down regulated when compared with the WA07 line (Fig. 2B and Table 1). Among these differentially expressed genes, *ENO 1* and *ENO 3* were downregulated (2.02 and 1.98 fold, respectively, p<0.05) in AE IPSC relatively to the WA07 line, whereas the expression of these did not significantly vary for the IMR-90 IPSC line. Instead IMR-90 IPSC displayed up regulation of *BPGM* (3.82 fold, p<0.01), and *HK2* (3.15 fold, p<0.05) when compared to the WA07 line (Fig. 2B, Table 1). Both H7TF and IMR-90 lines showed down regulation of *ENO 3* (4.72 fold, p<0.05 and 4.73 fold, p<0.01 for H7TF and IMR-90, respectively) in comparison with the WA07 (Fig. 2B, Table 1). In addition, the IMR-90 line showed decreased expression of *PGM2* (2.38 fold, p<0.01) and up regulation of *ALDO A* (2.64 fold, p<0.001).

In terms of the pentose phosphate pathway seven genes were differentially expressed among all the lines when compared with the WA07 line. No differences were observed between the WA07 line and both the WA01 and AE IPSC lines. The IMR-90 IPSC line showed up regulation of *H6PD* (4.65 fold, p<0.01), *PRPS1* (2.18 fold, p<0.05), *PRPS2* (2.17 fold, p<0.01), *TKT* (2.67 fold, p<0.01) in comparison with the WA07 line. Both the H7TF and IMR-90 lines displayed increased expression of *G6PD* (1.96 fold, p<0.001 and 2.82 fold, p<0.001, for H7TF and IMR-90, respectively) *H6PD* (7.33, p<0.05 and 7.82, p<0.01, respectively), and decreased expression of *TKT* (2.13 fold, p<0.05 and 2.65 fold, p<0.05, respectively) in comparison to the WA07 line. In addition the IMR-90 line showed decreased expression of *PRPS1* (3.96 fold, p<0.05), *RPE* (2.50 fold, p<0.05) and *RPIA* (2.52 fold, p<0.05).

Regarding the TCA cycle signature we observed that 17 genes were differentially expressed when compared to the WA07 line (Fig. 2D, Table 1). Again no significant differences were observed between the two hESC lines. When compared to the WA07 line, the AE IPSC line displayed decreased expression of *DLD* (1.98 fold, p<0.05) (Fig. 2C). The IMR-90 IPSC line showed increased expression of *ACO2* (2.09 fold, p<0.01), and *SUCLG2* (4.08 fold, p<0.05) when compared with the WA07 line (Fig. 2C). Both the H7TF and IMR-90 lines showed down regulation of *DLAT* (2.65 fold, p<0.01 and 4.65, p<0.001, for H7TF and IMR-90, respectively) and *IDH1* (6.34 fold, p<0.05 and 14.13 fold, p<0.01) for H7TF and IMR-90, respectively) when compared with the WA07 line (Fig. 2C, Table 1). In addition, the IMR-90 line demonstrated decreased expression of *CS* (2.21 fold, p<0.01), *DLD* (4.65 fold, p<0.001), *DLST* (2.22 fold, p<0.05), *IDH3B* (3.44 fold, p<0.01), *MDH1* (3.43 fold, p<0.05), *MDH1B* (6.52 fold, p<0.05), *PDHA1* (2.78 fold, p<0.01), *PDHB* (2.88, p<0.001), *SDHC* (1.96, p<0.01) and *SUCLA2* (2.52, p<0.05), and up regulation of *ACO 1* (2.13 fold, p<0.01).

With respect to genes involved in the regulation of TCA cycle activity two genes were differentially expressed among all lines and the WA07 line (Table 1). No significantly expressed genes were detected between WA07 line, and the WA01, AE IPSC and IMR-90 IPSC lines. Both differentiated lines showed down regulation of *PDP2* (3.52 fold, p<0.05 and 4.55 fold, p<0.01 for H7TF and IMR-90, respectively). In addition the IMR-90 displayed decreased expression of *PDK3* (5.32 fold, p<0.01).

### Mitochondrial contribution to the energy metabolism of human pluripotent stem cells and differentiated cells

**Figure 3:**
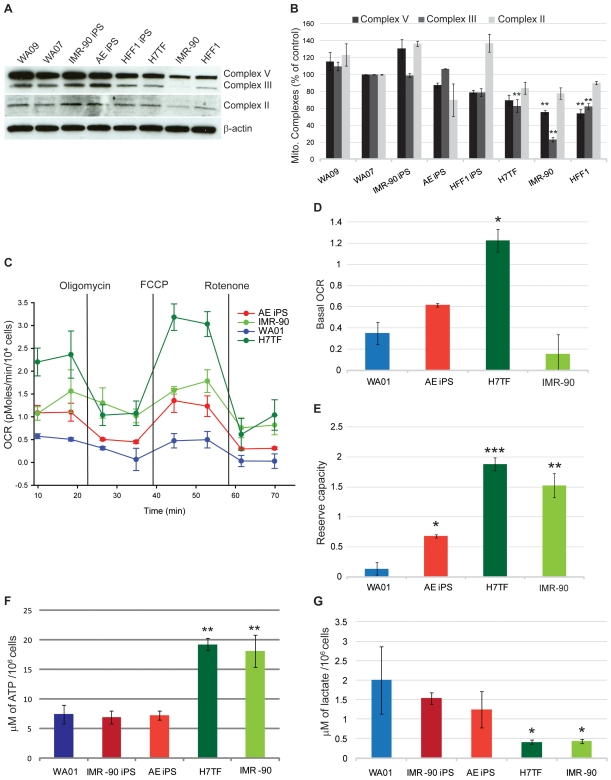
Mitochondrial contribution to the energetic metabolism of human pluripotent stem cells and differentiated cells. **A)** Western blotting analysis of mitochondrial complexes II, III and V. β-actin was used as a loading control. **B)** Quantification of mitochondrial complexes II, III and V protein levels relative to the WA07 line. **C)** Oxygen consumption rate (OCR) was determined by Seahorse XF24 analyzer. The three mitochondrial inhibitors were sequentially injected (after measurement points, 2, 4, and 6, as indicated) and the final concentrations of each were: oligomycin (1 µM); FCCP (300mM); rotenone (1 µM) **D)** Basal OCR (represents the mean of the first two measurements minus the mean of measurements 3 & 4). **E)** Reserve capacity (FCCP induced levels, measurements 5 & 6 minus the basal level). **F)** Intracellular ATP levels determined by HPLC. **G)** Lactate levels secreted to the media. The values are averages of three independent experiments. Statistical significance was determined by one-way ANOVA followed by Dunnet's multiple comparison test. Statistical significance was determined at P<0.05. Error bars: SEM.

To further understand mitochondrial contribution to the energy metabolism of pluripotent cells we analyzed protein expression of mitochondrial complexes II, III and V in pluripotent vs. differentiated somatic cells. We observed no statistical significant differences in mitochondrial complexes II, III and V between the various pluripotent lines. Intriguingly, we observed that pluripotent cells have higher protein levels of mitochondrial complexes, II, III and V than differentiated cells (Fig. 3A, B). Indeed, when compared to the WA07 line, mitochondrial complex V protein levels were decreased by 45% and 46% in IMR-90 and HFF1 fibroblasts, respectively (p<0.01 Fig. 3A,B). Furthermore, complex III protein levels were decreased by 76.8%, 38% and 27% in IMR-90, HFF1 and H7TF fibroblasts, respectively (p<0.01, Fig. 3A,B). Although, we observed a decreased expression trend for mitochondrial complex II in differentiated cells, there was not statistical significance.

In order to determine if the differences observed in mitochondrial complex expression could be translated in terms of higher mitochondrial activity in pluripotent cells we determine O_2_ consumption rate (OCR) using the Seahorse XF24 extracellular flux analyzer. We have used a pharmacological profiling approach, by combining the use of three mitochondrial inhibitors (rotenone, FCCP and oligomycin) and the Seahorse instrument as previously described by Qian and Van Houten [37]. The OCR response to the chemical compounds were analyzed in one hESC line (WA01), one IPSC line (AE) and two differentiated cell lines (H7TF and IMR-90).

The basal OCR (measure of OXPHOS) is the amount oxygen consumption that is linked to ATP synthesis in the mitochondria, and represents the mean basal levels of oxygen consumption minus the mean of the two values following oligomycin treatment (measurements 3 and 4). Oligomycin inhibits ATP synthase by binding to the oligomycin sensitivity-conferring protein (OSCP) at the F_0_ subunit of the ATP synthase. This binding blocks the proton conductance resulting in loss of electron transfer and O_2_ consumption. Addition of oligomycin resulted in decreased levels of OCR in all cell types (Fig. 3C). Nonetheless the kinetics and relative intensity of response varied among the various cell types with hESCs having the slowest and less pronounced response followed by IMR-90, AE IPSC and finally H7TF (Fig.3C).

**Figure 4:**
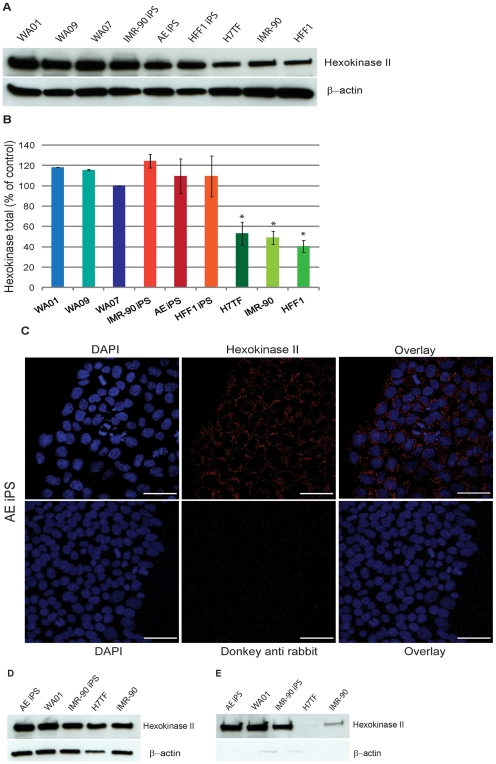
Hexokinase II expression in human pluripotent stem cells and differentiated cells. **A)** Western blotting analysis for hexokinase II protein levels. **B)** Quantification of Hexokinase II protein levels relative to the WA07 line. **C**–**E**) Hexokinase II protein levels in the cytoplasmic and mitochondrial fractions, respectively. β-actin was used as a loading control. Values are means of three independent experiments. Statistical significance was determined at p<0.05 by one-way ANOVA followed by Dunnet's multiple comparison test. Error bars: SEM

The H7TF cells showed higher basal O_2_ consumption rates when compared to the other cell types (Fig. 4C and 4E). The lowest basal OCR was observed for hESCs (WA01) and the differentiated line IMR-90 (Fig. 4C and 4E). The AE IPSC line displayed intermediate basal OCR levels between those found for the H7TF and WA01 line (Fig. 4C and 4D). Likewise to what was observed in glucose-related gene expression there were marked differences between the IMR-90 and H7TF with respect to their basal OCR. Because H7TF cells are derived from teratomas one could question whether the OCR levels of these cells are representative of other differentiated lines. In order to address that question we performed OCR measurements for a normal diploid human skin fibroblast line (NDHF). We observed that OCR levels in these cells are similar to those observed in H7TF rather to those observed in the IMR-90 cells (Fig.S1).

FCCP is a mitochondrial uncoupler that dissipates the proton gradient, and therefore uncouples electron transport and mitochondrial respiration from ATP synthesis. FCCP treatment has been shown to increase O_2_ consumption to its maximum in various cell types. In fact FCCP resulted in an increase of OCR levels in all cell types, with the most pronounced response being observed in H7TF, followed by IMR-90, AE IPS and WA01 cells (Fig. 3C). The difference between FCCP OCR and basal OCR is a good measure of respiratory capacity (also designed of reserve capacity) of these cells. Indeed we observed that the respiratory capacity of differentiated cells is higher than that observed for pluripotent lines (Fig.3E).

Rotenone is a mitochondrial inhibitor that prevents electron transfer from complex I to ubiquinone by blocking the ubiquinone binding site [38]. Treatment with rotenone decreased OCR levels in all cells types although H7TF fibroblasts showed a more pronounced response to the treatment. Overall our results suggest that both hESCs and IPSCs have lower reliance in OXPHOS when compared to the H7TF cells. Interestingly IMR-90 fibroblasts showed low basal O_2_ consumption rates, but when challenged with FCCP had the ability to increase OXPHOS rates to a similar extent to that observed in other differentiated cell types (H7TF and NDHF). In contrast both hESCs and IPSCs showed a considerably reduced response to FCCP than differentiated cells (H7TF and NDHF). These results suggest that mitochondria in pluripotent cells, although functional, present a low activity rate, and that the differences in mitochondrial content noted previously do not directly account for differences in activity. Indeed we observed that mitochondria membrane potential is lower in hESCs when compared to their differentiated counterparts (data not shown).

Total ATP levels are originated both from anaerobic and aerobic respiration. In order to determine intracellular ATP levels in human pluripotent stem cells and differentiated cells we performed HPLC (Fig. 3F). Our results showed that somatic cells have elevated intracellular ATP levels when compared to differentiated cells. In addition we measure lactate secretion to media (a good measure for glycolysis that does not add to the TCA cycle) in human pluripotent stem cells and differentiated cells (Fig.3G). As expected human pluripotent cells displayed higher lactate levels than differentiated cells. Overall these results suggest that human pluripotent stem cells rely mostly on glycolysis to meet their energy demands.

### Hexokinase II expression in human pluripotent stem cells and differentiated cells

Mammalian tissues harbor four hexokinases isoforms (I–IV) with particular kinetics and tissue specificity. Both hexokinase I and II have an N-terminal hydrophobic domain that allows binding to the outer mitochondrial membrane. Pedersen and colleagues have shown that mitochondrial-bound hexokinase II is responsible for the high glycolytic profile of rat hepatoma cells [26], [27]. In order to determine if hexokinase II could play a role in the maintenance of high glycolytic rates in pluripotent cells we performed western blotting analysis for hexokinase II in three hESC lines (WA01, WA07 and WA09), three IPSC lines (IMR-90 IPSC, AE IPS and HFF1 IPSC) three differentiated lines (H7TF, IMR-90 and HFF1). We observed that pluripotent cells overexpress hexokinase II when compared to differentiated cells (Fig 4A, B). We next isolated the cytoplasmic and mitochondrial fractions of hESCs (WA01 line), IPSCs (AE IPS and IMR-90 IPS) and fibroblasts cells (H7TF and IMR-90) and determined hexokinase protein levels in both fractions (Fig. 4C, D, respectively). We observed that hexokinase II in differentiated cells is preferentially localized in the cytoplasm, whereas pluripotent cells display high levels of hexokinase II in both cytoplasmic and mitochondrial fractions. Binding of hexokinase II to the outer mitochondrial membrane can enhance mitochondrial metabolism in two ways; first when bound to the outer mitochondrial membrane, hexokinase escapes the inhibitory effect of its product glucose 6-phosphate; second mitochondrial binding allows hexokinase II to gain access to ATP that permeates the voltage dependent anion channel [39], [40].

### PDH complex regulation in human pluripotent stem cells and differentiated cells

**Figure 5:**
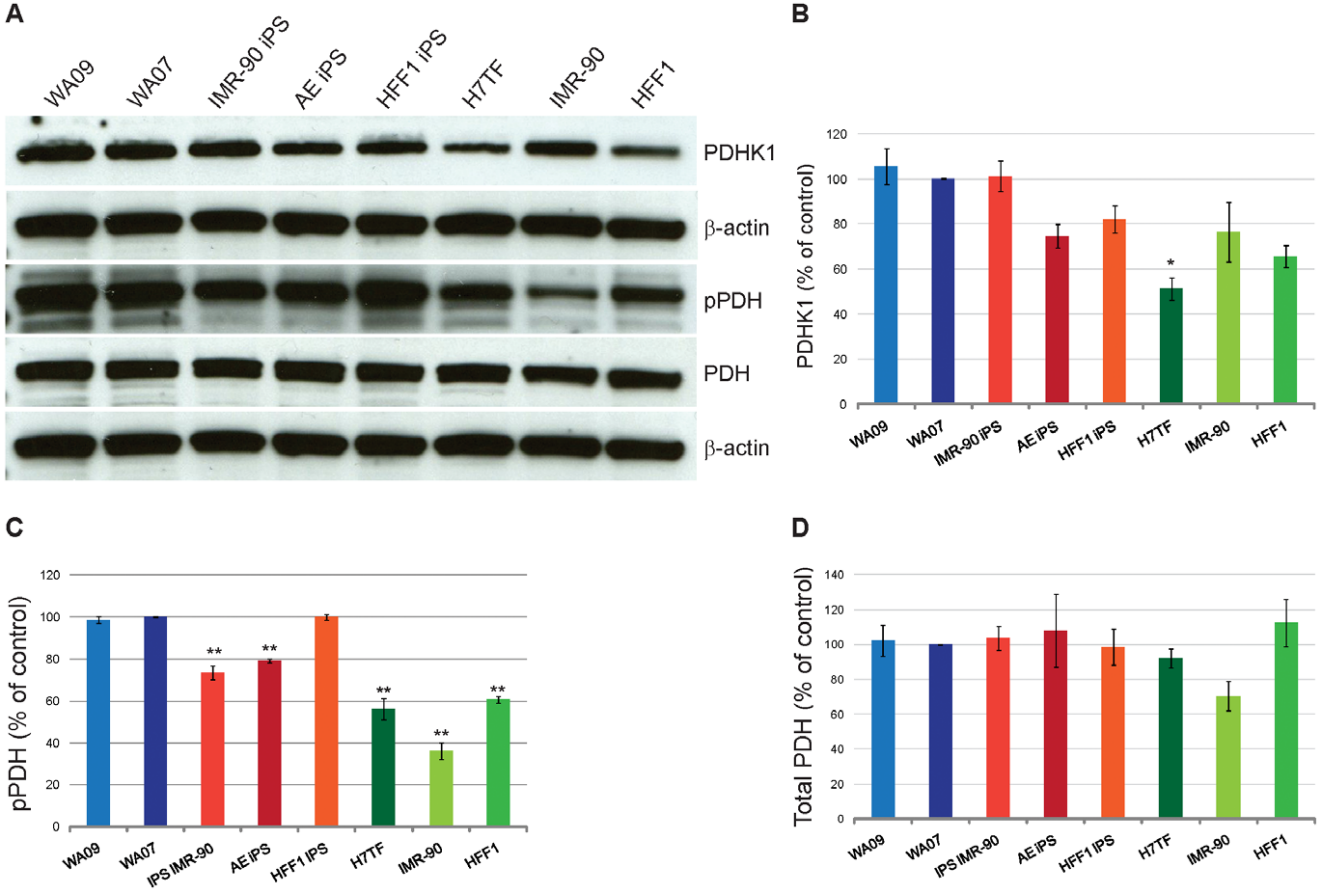
Pyruvate dehydrogenase regulation in human pluripotent stem cells and differentiated cells. **A)** Western blotting analysis of PDHK1, pPDH and total PDH protein levels. β-actin was used as a loading control. **B**–**D)** Protein levels quantification relative to the Wa07 line of PDHK1, pPDH and PDH, respectively. Values are means of three independent experiments. Statistical significance was determined at p<0.05 by one-way ANOVA followed by Dunnet's multiple comparison test. Error bars: SEM Abbreviations: PDHK1, pyruvate dehydrogenase kinase one; pPDH: phospho Ser ^293^ pyruvate dehydrogenase subunit E1α; PDH: pyruvate dehydrogenase.

Key metabolic regulation in terms of glycolysis versus OXPHOS involves the PDH complex. We therefore analyzed PDHK1, phospho PDH and total PDH protein in pluripotent stem cells and differentiated cells. We observed that PDHK1 protein levels are up regulated in pluripotent cells when compared to differentiated cells (Fig.5 A, B). Statistical analysis revealed that there are no significant differences between the WA07 hESC line and the other pluripotent lines (Fig. 5B). Although, there was a decrease in PDHK1protein levels between the WA07 line and both H7TF and HFF1 fibroblast lines (49% and 34% decrease for H7TF and HFF1, respectively; p<0.05) no significant differences were observed for the IMR-90 line (Fig. 5B). Interestingly, we did not observe an increase in *PDK1* gene expression in pluripotent vs. differentiated cells (Fig. S2 and Table S1). This result suggests that PDHK1 protein stabilization differs between pluripotent and differentiated cells. Consistent with higher PDHK1 protein levels, pluripotent cells also displayed higher phospho PDH levels when compared to differentiated cells (Fig.5 A and C). All three differentiated lines showed lower phospho PDH protein when compared with the hESC line WA07 ( 44%, 64% and 39% decrease for H7TF, IMR-90 and HFF1 fibroblast, respectively; p<0.01; Fig. 5C). Interestingly, both AE and IMR-90 IPSCs showed lower levels of phospho-PDH than the WA07 line (20% and 26% decrease for AE IPSCs and IMR-90 IPSCs, respectively; p<0.01; Fig. 5C). No significant differences were found for total PDH levels. These results suggest that the lower mitochondrial activity observed in pluripotent cells might, at least partially, be attributed to high levels of phospho PDH. Our results further suggest that besides PDHK1 other PDHKs are regulating the activity of the PDH complex in these cells, as no significant differences were observed for the PDHK levels in IMR-90 cells but these cells displayed higher levels of phospho PDH.

## Discussion

In this study we sought to understand the energetic metabolism of pluripotent stem cells and their differentiated counterparts. We started by looking at mitochondrial morphology and localization in human pluripotent stem cells (both hESCs and IPSCs), and somatic cells. In agreement with previous reports our results demonstrate that mitochondria in hESCs appear immature with fewer cristae, and low electron density matrix, when compared to differentiated somatic cells [16], [20]. Interestingly IPSCs displayed a mixed mitochondrial phenotype that still resembles the somatic cells of origin and is not quite that of ESCs.. Analyzing distinct mitochondrial features during the reprogramming process and how they compare with the acquisition and stabilization of pluripotency characteristics would therefore be of interest, and might provide novel insights into mitochondrial activity and metabolic shifts during reprogramming.

Glucose-related gene expression analysis revealed that both hESC lines analyzed in this study have identical glucose-related metabolism signatures. In contrast IPSCs are not identical to hESCs, although they cluster together, rather than with their somatic counterparts. Interestingly both IPSC lines analyzed in this study showed different metabolic gene expression signatures, with IMR-90 IPSCs having higher transcripts levels. It should be noted that that the cocktail of transcription factors used for the reprogramming of both lines differ [1]. However recent papers [10] established a genome-wide map for DNA methylation and gene expression for a considerable number of IPSC lines generated using several somatic cells and various methodologies, demonstrating some cell-line differences in gene expression that are probably not due to a single factor. It would be interesting to determine if these differences are also reflected in terms of functional metabolic studies.

Regarding the glycolytic signature of human pluripotent stem cells and somatic cells we observed that H7TF fibroblasts displayed similar glycolytic signature to their pluripotent counterpart the WA07 hESC line, with only four genes significantly downregulated. Interestingly the IMR-90 fibroblasts displayed 10 genes differentially expressed (six genes significantly downregulated and four genes up regulated when compared to the WA07).

Cell division requires not only ATP but biosynthetic precursors derived from glycolysis, the pentose phosphate pathway and the TCA cycle. For instance, glucose 6-phosphate can be diverted from the glycolytic pathway to the pentose phosphate pathway in order to generate ribose 5-phophate for *de novo* nucleotide synthesis. The pentose phosphate pathway can be divided in two major branches, an oxidative branch and a non-oxidative branch [41]. Our gene expression analysis demonstrated that both *G6PD* and *H6PD* are significantly up regulated in differentiated cells. The enzymes encoded by these genes catalyze the first step in the oxidative branch of the pentose phosphate pathway that involves the conversion of glucose 6-phosphate to 6-phosphogluconate with concomitant production of NADPH. Besides its role as a cofactor in many biosynthetic reactions, NADPH is essential to regenerate reduced glutathione from glutathione disulfide [42]. Hence *G6PD* and *H6PD* transcripts levels may reflect high requirement for NADPH, either as reducing agent for biosynthetic pathways or instead as a cofactor in the cell antioxidant defense. The latter hypothesis is supported by the fact that differentiated cells have a higher mitochondrial activity and therefore a higher potential for ROS production. Furthermore, we observed that human pluripotent cells have higher expression levels of genes related to the non-oxidative branch, such as *TKT*, and *RPIA.* These results suggest that human pluripotent cells preferentially use the non-oxidative branch of the pentose phosphate pathway in order to obtain ribose-5 phosphate which is important for nucleotide synthesis. A similar pattern has been previously described for cancer cells [43], [44], [45].

Interestingly, the TCA cycle signature revealed that most of these genes are downregulated in differentiated cells, when compared to pluripotent lines. Besides its role in oxidative catabolism of carbohydrates and fatty acids, the TCA cycle provides precursors for many biosynthetic pathways, including precursors for amino acid and nucleotide synthesis. It is possible that up regulation of TCA cycle-related genes in human pluripotent stem cells is related to the high proliferative rates and concomitant need for nucleotides rather than need for reducing agents for mitochondrial activity. This issue needs to be explored further. Regardless, higher levels of genes encoding TCA cycle proteins might be indicative of increased electron transport capacity and cellular respiration. Consistent with increased level of genes encoding proteins of the TCA cycle we observed that pluripotent lines have higher levels of mitochondrial electron transport complexes than differentiated cells. The transcription factor c-Myc is crucial for the maintenance of embryonic stem cell self-renewal and its ectopic expression allows the reprogramming of somatic cells to an embryonic stem cell like state [1], [3]. In addition this transcription factor has been previously described to up regulate the expression of several glycolytic enzymes as well as to promote mitochondrial biogenesis [46], [47], [48], [49]. Hence higher levels of complex II, III and V may be due to higher c-myc levels in pluripotent cells and may not be representative of high mitochondrial activity. In accordance with this hypothesis our OCR results showed that pluripotent lines consume lower levels of O_2_ than the differentiated line H7TF. However, IMR-90 fibroblasts displayed similar basal OCR levels than those found in pluripotent lines. These results could be related to the fetal origin of the IMR-90 fibroblasts, as the fetal lung is exposed to a rather hypoxic environment [50]. In agreement with this notion, when we induced mitochondrial respiration by the use of FCCP (reserve capacity) IMR-90 fibroblasts could increase OCR to similar levels to those found in H7TF. Importantly, human pluripotent stem cells did not display a strong response to FCCP treatment suggesting that aerobic respiration in these cells is somewhat impaired. Consistent with less active mitochondria, human pluripotent cells showed reduced ATP levels when compared with differentiated cells. In addition these cells secreted higher lactate levels indicative of higher glycolytic rates. Thus despite higher levels of genes encoding proteins from the TCA cycle and higher content in mitochondrial electron transport chain complexes, human pluripotent cells seem to have lower overall OXPHOS activity. It remains to be established why this is the case, but our data suggests some possibilities.

Hexokinase II catalyzes the first reaction of glycolysis and it has been previously demonstrated that its expression is restricted to the inner cell mass of the blastocyst, and that HK2 knock down results in mouse embryonic lethality around E.D. 7.5 [51], [52]. Although we did not observe significant differences at mRNA level, the total protein levels of hexokinase II were significantly elevated in human pluripotent stem cells. These results suggest that hexokinase II protein stabilization might be increased in human pluripotent lines. Higher levels of mitochondrial hexokinase II can be advantageous for glycolytic metabolism in two ways: first binding of hexokinase II the outer mitochondrial membrane allows this enzyme to escape inhibition by its product glucose-phosphate; and second it allows the enzyme to gain access to newly synthesized ATP required for the phosphorylation of glucose [39], [40]. In addition, hexokinase II plays a key role in the prevention of cell death by binding to VDAC, therefore representing a link between glucose metabolism and apoptosis [53], [54]. Hence it is possible that hexokinase II plays a role in preventing human pluripotent stem cell apoptosis as well.

On the other hand, the PDH complex is a crucial step in regulation of metabolism since it constitutes the link between anaerobic metabolism and the TCA cycle. Phosphorylation of the PDH E1α subunit leads to inactivation of the PDH complex and consequently results in lower levels of acetyl CoA to enter the TCA cycle. Tellingly, pluripotent lines have higher levels of phosphorylated PDH E1α. Phosphorylation of PDH complex can be carried out by four PDHKs and in this study we analyzed PDHK1 expression levels. Although we did not observe an increase in PDHK1 gene expression in pluripotent lines when compared to differentiated cells we did observe an increase in PDHK1 protein levels in pluripotent cells. These results suggest that PDHK1 protein stability differs between pluripotent and differentiated cells. Papandreou and colleagues have previously demonstrated that under hypoxic conditions hypoxia inducible factor (HIF-1) up regulation resulted in increased expression of PDHK1 leading to inactivation of the PDH complex. This in turn resulted in reduced substrate availability to enter the TCA cycle and led to decreased oxidative phosphorylation [55]. HIF-1a might therefore be considered a good target for future work, given that we observed that some of its targets are involved in the maintenance of this glycolytic profile.

Overall our results demonstrate that human pluripotent cells have a greater reliance on glycolysis than differentiated cells. In addition our study suggests that this can be mediated by increasing hexokinase II levels and inactivation of the PDH complex. Interestingly these metabolic strategies involving features of anaerobic metabolism under normoxia are also found in many types of tumor cells [34], [56], and parallel assays in both pluripotent and tumor lines would be extremely interesting Importantly we demonstrate that despite the fact that both hESCs and IPSCs rely on glycolysis, these cell types are not identical in terms of glucose-related gene expression, mitochondrial morphology, and O_2_ consumption. This suggests that IPSC somatic cell reprogramming to IPSC may result in differences at the metabolic level, when compared to the pluripotent standard of hESC. While epigenetic and transcriptomic differences have been mentioned above [9], [10] other significant genetic changes in IPSCs when compared to hESCs and differentiated cells were also recently described [11], [12], including higher mutation rates and copy number variation. Interestingly, recent data suggests that IPSCmitochondria retain significant developmental plasticity upon IPSC generation, and somatic cell re-differentiation. [57].

In addition we also observe that not all the differentiated lines display the same metabolic profile and this might have an impact in the reprogramming efficiency of various somatic cell types, and on the characteristics of differentiated cells obtained from different IPSC lines. These results stress the importance of the complete characterization of these cell lines, and suggest that there may be other links to tumor cells besides those already described.

## Supporting Information

Figure S1 **Comparison of OCR in two differentiated cell lines.** A**)** Oxygen consumption rate (OCR) was determined by Seahorse XF24 analyzer for IMR-90 and NHDF lines, the former also listed in Figure 3. The mitochondrial inhibitors were sequentially injected at specific time points as illustrated in the figure. **B)** Basal OCR (represents the mean of the first three measurements minus the mean of measurements 4, 5 & 6). **E)** Reserve capacity (FCCP induced levels, measurements 7, 8 & 9 minus the basal level). Measurements show that NHDF cells show higher values than IMR-90 cells, similarly to what is described for the other differentiated cell line (H7TF) listed in Figure 3
(TIF)

Figure S2 **p-values for the glucose metabolism gene expression array in human pluripotent stem cells vs. differentiated cells**. Statistical analysis was performed using SABiosciences online software and Student's t test was applied. Significance was determined at p<0.05.
(PDF)

Table S1 **Average Ct values for gene expression**. Differences were calculated using the -ΔΔCt method and gene expression was normalized to the housekeeping gene β-actin. Average Ct values were determined using online software provided by SABiosciences.
(PDF)
