# Supplementary figures and images for: Energy Metabolism in Human Pluripotent Stem Cells and Their Differentiated Counterparts
Source: PLoS One. 2011 Jun 17;6(6):e20914. doi: 10.1371/journal.pone.0020914 (PMC3117868; doi:10.1371/journal.pone.0020914)

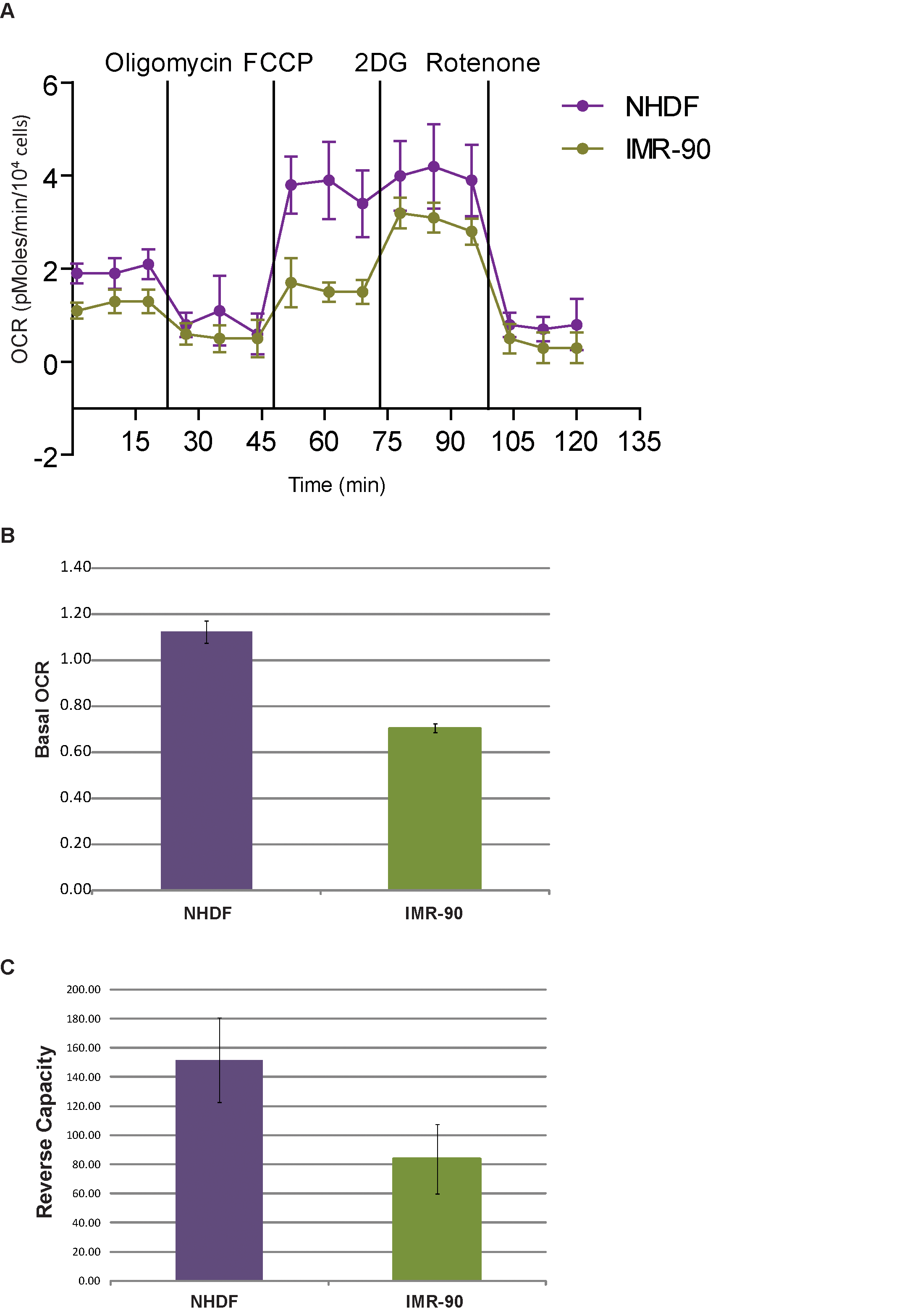

Supplement: Figure S1 — Comparison of OCR in two differentiated cell lines. A) Oxygen consumption rate (OCR) was determined by Seahorse XF24 analyzer for IMR-90 and NHDF lines, the former also listed in Figure 3. The mitochondrial inhibitors were sequentially injected at specific time points as illustrated in the figure. B) Basal OCR (represents the mean of the first three measurements minus the mean of measurements 4, 5 & 6). E) Reserve capacity (FCCP induced levels, measurements 7, 8 & 9 minus the basal level). Measurements show that NHDF cells show higher values than IMR-90 cells, similarly to what is described for the other differentiated cell line (H7TF) listed in Figure 3 (TIF) [file pone.0020914.s001.tif]
