# Supplementary material for: Energy Metabolism in Human Pluripotent Stem Cells and Their Differentiated Counterparts
Source: PLoS One. 2011 Jun 17;6(6):e20914. doi: 10.1371/journal.pone.0020914 (PMC3117868; doi:10.1371/journal.pone.0020914)
Supplement: Figure S2 — p-values for the glucose metabolism gene expression array in human pluripotent stem cells vs. differentiated cells. Statistical analysis was performed using SABiosciences online software and Student's t test was applied. Significance was determined at p<0.05. (PDF) [file pone.0020914.s002.pdf]

p values					
Genes	**WA01**	**AE iPS**	**IMR-90 iPS**	**H7TF**	**IMR-90**
**ACLY**	0.107296	0.234368	0.061387	0.393416	0.246977
**ACO1**	0.399131	0.079385	0.17169	0.083256	0.001386
**ACO2**	0.211968	0.089029	0.006734	0.15926	0.006435
**AGL**	0.716072	0.005425	0.192293	0.003653	0.000895
**ALDOA**	0.193238	0.116809	0.00108	0.086865	0.00002
**ALDOB**	0.606445	0.046384	0.011521	0.331536	0.339268
**ALDOC**	0.106059	0.07849	0.003951	0.108363	0.652673
**BPGM**	0.419129	0.088599	0.032134	0.154385	0.139919
**CS**	0.598438	0.052026	0.45459	0.548219	0.003514
**DLAT**	0.23882	0.369543	0.531728	0.001807	0.000145
**DLD**	0.860033	0.027963	0.493081	0.671359	0.004943
**DLST**	0.628177	0.019154	0.018305	0.726826	0.036226
**ENO1**	0.143852	0.219918	0.859918	0.014508	0.040667
**ENO2**	0.11791	0.075848	0.556327	0.089909	0.627736
**ENO3**	0.186648	0.012241	0.180077	0.006147	0.003549
**FBP1**	0.126317	0.914908	0.010103	0.066292	0.064231
**FBP2**	0.114863	0.003239	0.006201	0.681758	0.001413
**FH**	0.189939	0.945714	0.123065	0.242483	0.105099
**G6PC**	0.606445	0.046384	0.011521	0.331536	0.339268
**G6PC3**	0.164792	0.949088	0.208358	0.531407	0.277338
**G6PD**	0.222918	0.12939	0.009571	0.000621	0.000546
**GALM**	0.271864	0.216248	0.607923	0.644125	0.008806
**GBE1**	0.175241	0.182796	0.401636	0.059971	0.003255
**GCK**	0.606445	0.046384	0.011521	0.331536	0.339268
**GPI**	0.293344	0.102082	0.084181	0.112545	0.028433
**GSK3A**	0.119705	0.144921	0.083618	0.408039	0.061973
**GSK3B**	0.434825	0.208689	0.086282	0.844473	0.094813
**GYS1**	0.560387	0.305042	0.073519	0.361399	0.006032
**GYS2**	0.606445	0.046384	0.011521	0.331536	0.339268
**H6PD**	0.536777	0.772588	0.001435	0.022755	0.005125
**HK2**	0.113544	0.67292	0.033784	0.292525	0.951775
**HK3**	0.606445	0.046384	0.011521	0.331536	0.339268
**IDH1**	0.112953	0.38673	0.274658	0.0133	0.009346
**IDH2**	0.109272	0.7786	0.031926	0.983299	0.759598
**IDH3A**	0.291665	0.144282	0.699497	0.35153	0.107709
**IDH3B**	0.126595	0.753791	0.063038	0.271417	0.002515
**IDH3G**	0.127383	0.888987	0.020491	0.160844	0.22335

**MDH1**	0.136175	0.888013	0.220693	0.24667	0.032701
**MDH1B**	0.162897	0.520214	0.113153	0.126206	0.019708
**MDH2**	0.741719	0.076133	0.030813	0.960944	0.012102
**OGDH**	0.20949	0.383583	0.033559	0.773533	0.003039
**PC**	0.10867	0.486329	0.070307	0.698611	0.144088
**PCK1**	0.606445	0.046384	0.011521	0.331536	0.339268
**PCK2**	0.10813	0.884791	0.082829	0.167213	0.250898
**PDHA1**	0.770854	0.041691	0.395379	0.045818	0.00137
**PDHB**	0.529193	0.012731	0.486834	0.018482	0.000693
**PDK1**	0.125991	0.108717	0.306787	0.106219	0.317454
**PDK2**	0.136513	0.180319	0.736911	0.114486	0.274814
**PDK3**	0.168418	0.065322	0.033078	0.14104	0.008899
**PDK4**	0.809515	0.01443	0.012961	0.436308	0.305641
**PDP2**	0.128547	0.403003	0.720828	0.015999	0.008756
**PDPR**	0.205352	0.658809	0.959175	0.095329	0.064798
**PFKL**	0.249233	0.313119	0.224002	0.346346	0.052093
**PGAM2**	0.525487	0.744314	0.893737	0.031701	0.055437
**PGK1**	0.522596	0.075003	0.045169	0.125637	0.0107
**PGK2**	0.606445	0.046384	0.011521	0.331536	0.339268
**PGLS**	0.854679	0.021219	0.088223	0.276943	0.975412
**PGM1**	0.118692	0.108458	0.557582	0.443997	0.255193
**PGM2**	0.314817	0.234121	0.248629	0.699586	0.002456
**PGM3**	0.219734	0.069024	0.263436	0.527696	0.591157
**PHKA1**	0.117699	0.046949	0.322249	0.283362	0.011132
**PHKB**	0.759246	0.227841	0.905398	0.490711	0.052339
**PHKG1**	0.857115	0.004105	0.098563	0.538787	0.712081
**PHKG2**	0.184464	0.330626	0.305181	0.085937	0.09916
**PKLR**	0.108278	0.219514	0.664814	0.672789	0.653998
**PRPS1**	0.245115	0.126676	0.019419	0.128065	0.018916
**PRPS1L1**	0.606445	0.046384	0.011521	0.331536	0.339268
**PRPS2**	0.831567	0.056076	0.004843	0.465994	0.305017
**PYGL**	0.12035	0.182944	0.63261	0.469592	0.834941
**PYGM**	0.105912	0.300795	0.072922	0.163273	0.161997
**RBKS**	0.119577	0.899118	0.072344	0.913592	0.133126
**RPE**	0.121207	0.471102	0.264923	0.753951	0.037438
**RPIA**	0.300618	0.263414	0.010121	0.012123	0.000578
**SDHA**	0.518039	0.053203	0.04001	0.445353	0.210469
**SDHB**	0.835404	0.151871	0.250403	0.557484	0.01685
**SDHC**	0.232507	0.009183	0.168632	0.033296	0.002187
**SDHD**	0.415562	0.059517	0.463437	0.475622	0.011049

**SUCLA2**	0.340556	0.193395	0.129939	0.172629	0.014183
**SUCLG1**	0.328695	0.025023	0.153357	0.198642	0.045376
**SUCLG2**	0.106127	0.465278	0.01718	0.621296	0.237038
**TALDO1**	0.61781	0.043175	0.123504	0.065059	0.003374
**TKT**	0.152685	0.541674	0.001309	0.020472	0.010954
**TPI1**	0.304767	0.060271	0.187325	0.004987	0.014742
**UGP2**	0.140358	0.583771	0.349007	0.009063	0.00436
