# Supplementary material for: Energy Metabolism in Human Pluripotent Stem Cells and Their Differentiated Counterparts
Source: PLoS One. 2011 Jun 17;6(6):e20914. doi: 10.1371/journal.pone.0020914 (PMC3117868; doi:10.1371/journal.pone.0020914)
Supplement: Table S1 — Average Ct values for gene expression. Differences were calculated using the -ΔΔCt method and gene expression was normalized to the housekeeping gene β-actin. Average Ct values were determined using online software provided by SABiosciences. (PDF) [file pone.0020914.s003.pdf]

AVG Ct						
**GENES**	**WA01**	**AE iPS**	**IMR-90 iPS**	**H7TF**	**IMR-90**	**WA07**
**ACLY**	20.68	20.37	21.19	22.38	22.74	21.62
**ACO1**	23.17	23.06	24.08	23.88	22.12	22.93
**ACO2**	23.45	23.04	23	23.1	24.15	23.22
**AGL**	23.83	24.28	24.82	25.7	25.94	23.71
**ALDOA**	19.31	18.79	19.37	19.37	18.35	19.47
**ALDOB**	30	30	30	30	30	30
**ALDOC**	24.26	23.63	24.13	28.1	25.08	25.22
**BPGM**	23.51	23.53	23.9	22.74	23.13	23.54
**CS**	20.96	21.07	21.66	21.56	22.4	20.98
**DLAT**	21.73	21.69	22.56	23.56	24.35	21.85
**DLD**	25.68	26.16	26.64	26.24	27.34	25.65
**DLST**	22.62	22.38	23.08	23.05	23.99	22.56
**ENO1**	17.98	20.79	19.01	19.29	19.19	18.2
**ENO2**	23.54	23.28	23.3	24.09	22.77	22.73
**ENO3**	24.58	25.3	25.22	27.32	27.3	24.78
**FBP1**	25.91	26.32	25.51	29.94	30	26.87
**FBP2**	25.22	27.38	28.23	25.69	30	25.51
**FH**	21.26	21.18	21.68	21.38	22.58	21.68
**G6PC**	30	30	30	30	30	30
**G6PC3**	23.76	24.06	24.62	25.43	26.11	24.85
**G6PD**	25.93	25.35	26.14	25.42	24.87	26.09
**GALM**	25.56	25.16	26.04	25.49	26.3	25.32
**GBE1**	23.45	23.7	24.32	21.73	20.86	23.67
**GCK**	30	30	30	30	30	30
**GPI**	20.61	20.79	21.21	21.51	21.3	20.7
**GSK3A**	24.25	23.72	24.73	25.21	25.57	24.59
**GSK3B**	22.29	22.29	22.55	22.72	23.17	22.42
**GYS1**	23.88	23.8	23.83	24.01	22.92	23.97
**GYS2**	30	30	30	30	30	30
**H6PD**	27.43	26.44	25.78	24.58	24.47	27.15
**HK2**	25.2	25.33	25.53	27.87	26.4	26.34
**HK3**	30	30	30	30	30	30
**IDH1**	19.58	19.95	21.6	23.04	24.17	20.07
**IDH2**	23.13	23.21	23.48	23.87	23.73	23.58
**IDH3A**	23.04	22.84	23.36	22.58	23.51	22.66
**IDH3B**	21.91	21.75	22.43	22.89	24.22	22.15
**IDH3G**	23.44	23.25	23.66	23.38	23.78	23.76
**MDH1**	19.31	19.34	20.12	20.8	21.97	19.91

**MDH1B**	26.41	26.79	27.01	29.02	30	27.01
**MDH2**	20.01	19.68	20.02	20.19	20.67	19.89
**OGDH**	23.31	22.91	23.49	23.76	24.54	23.49
**PC**	25.16	25.22	25.72	26.85	25.39	26.56
**PCK1**	30	30	30	30	30	30
**PCK2**	22	22.25	22.61	24.95	24.38	23.01
**PDHA1**	21.59	21.61	22.09	22.36	23.22	21.46
**PDHB**	20.85	20.94	21.39	21.81	22.5	20.7
**PDK1**	24.59	24.85	23.69	25.67	23.06	22.36
**PDK2**	28.48	27.96	28.56	27.18	29.32	27.86
**PDK3**	22.91	23.56	22.41	23.68	25.05	22.36
**PDK4**	30	30	30	30	27.9	29.91
**PDP2**	24.22	24.53	25.51	26.7	27.05	24.58
**PDPR**	23.85	23.65	25.12	26.09	26.02	24.36
**PFKL**	23.34	22.73	23.15	22.7	22.34	22.81
**PGAM2**	26.88	26.29	27.52	28.77	28.05	26.67
**PGK1**	19.71	19.74	19.68	20.22	18.81	19.49
**PGK2**	30	30	30	30	30	30
**PGLS**	22.37	22.44	22.51	21.94	22.55	22.27
**PGM1**	23.49	23.23	22.34	22.2	22.52	21.52
**PGM2**	22.79	22.72	23.4	23.32	24.43	22.89
**PGM3**	22.35	22.73	23.11	22.59	23.08	22.46
**PHKA1**	26.53	26.35	26.56	26.98	28.01	25.99
**PHKB**	22.92	22.86	23.59	22.81	23.64	22.78
**PHKG1**	28.99	28.95	29.4	29	29.43	28.9
**PHKG2**	26.3	25.81	27.16	27.83	27.7	26.69
**PKLR**	28.62	30	30	30	30	29.56
**PRPS1**	21.55	21.3	20.78	22	23.33	21.06
**PRPS1L1**	30	30	30	30	30	30
**PRPS2**	23.5	22.63	23.18	23.55	23.88	23.46
**PYGL**	24.54	23.62	23.57	23.8	23.15	23.19
**PYGM**	26.63	26.41	27.29	30	30	27.8
**RBKS**	25.92	26.22	26.48	27.15	28.11	26.69
**RPE**	21.05	21.3	21.94	21.99	23.11	21.5
**RPIA**	23.7	25.21	23.83	24.72	25.39	23.78
**SDHA**	21.8	24.79	22.08	21.86	22.42	21.83
**SDHB**	21.52	21.38	21.94	21.64	22.5	21.49
**SDHC**	24.67	24.95	25.41	25.79	25.96	24.71
**SDHD**	24.18	24.28	24.92	24.77	25.29	24.25
**SUCLA2**	21.71	21.98	22.26	22.88	23.53	21.91

**SUCLG1**	23.38	23.42	24.26	23.02	23.89	23.09
**SUCLG2**	23.15	23.17	23.21	24.09	23.65	24.4
**TALDO1**	20.85	20.56	21.2	21.63	21.58	20.73
**TKT**	19.86	19.48	19.56	21.53	21.82	20.14
**TPI1**	19.43	19.05	19.82	20.25	19.08	19.21
**UGP2**	18.63	18.69	19.54	21.4	22.39	19
